# Supplementary material for: MicroRNA-15a-5p mediates abdominal aortic aneurysm progression and serves as a potential diagnostic and prognostic circulating biomarker
Source: Commun Med (Lond). 2025 Jun 6;5:218. doi: 10.1038/s43856-025-00892-w (PMC12144292; doi:10.1038/s43856-025-00892-w)
Supplement: Supplementary file 2 — Supplementary Information [file 43856_2025_892_MOESM2_ESM.pdf]

Supplemental Figure 1.

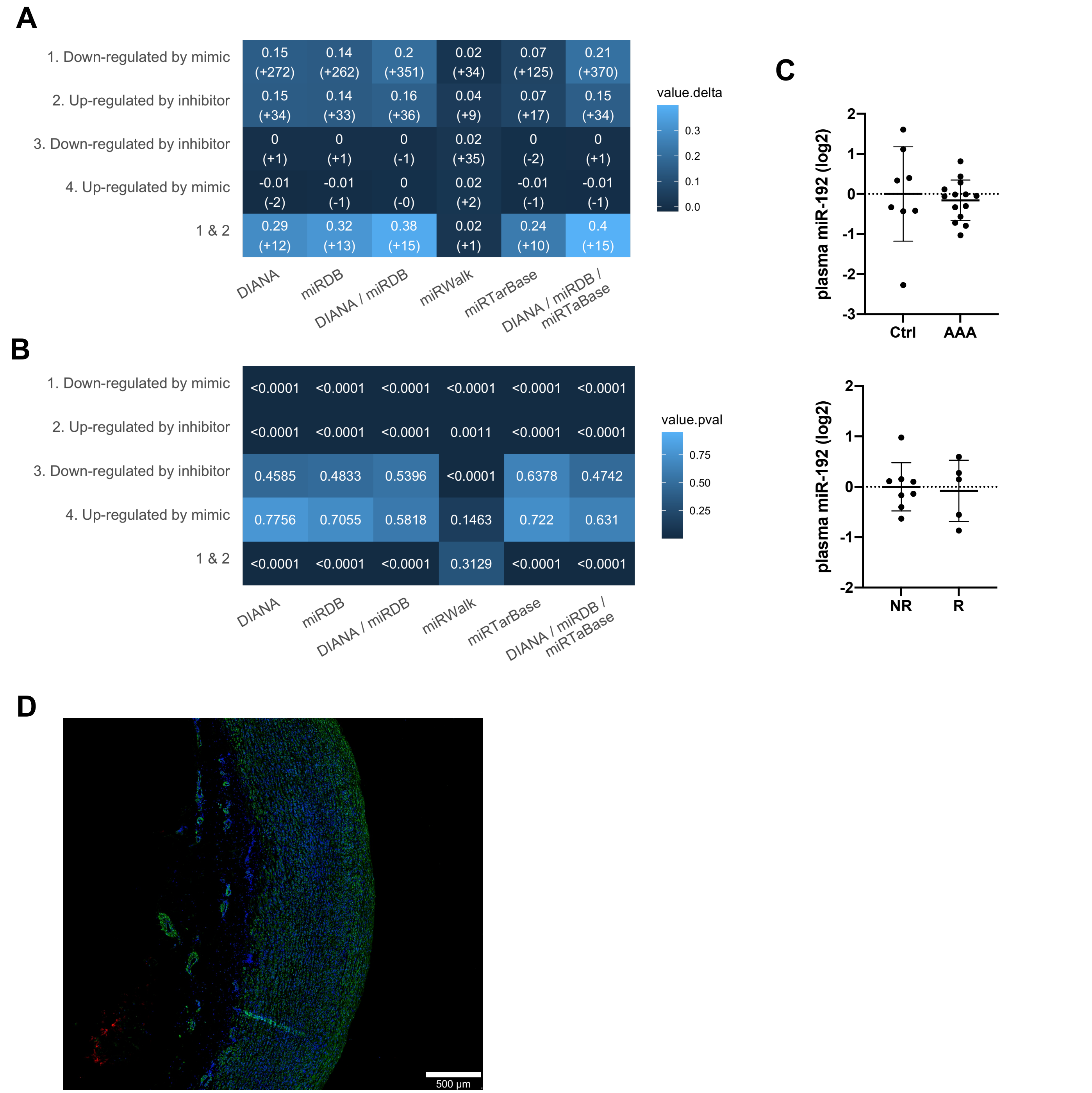

A. Fraction of predicted or confirmed miR-15a target genes significantly down-regulated (FDR<0.05, fold change (log2) < -1) upon miR-15a mimic treatment in hAoSMCs (corrected for expected overlap due to background signal). **B.** P-values from hypergeometric test (one-tailed Fisher's exact test) indicating significance of overlap between list of predicted or confirmed miR-15a target genes and list of significantly down-regulated (FDR<0.05, fold change (log2) < -1) upon miR-15a mimic treatment in hAoSMCs. **C.** Top: Expression of miR-192 in plasma of AngII model mice (n=13) and saline controls (n=8). Bottom: Expression of miR-192 in plasma of AngII model mice whose aortas were either ruptured/dissected (R; n=5) or non-ruptured/non-dissected (NR; n=8).
D. Fluorescent *in situ* hybridization with a scrambled miRNA control probe (red; Alexa647), immunofluorescence of α-SMA (SMA; green; Alexa555), fluorescently stained nuclei (DAPI) in mouse aortic tissue from the PPE model. Limited unspecific hybridization can be seen in the adventitial layer, but not in the SMA-positive medial layer, in contrast to images shown in Fig 1G). Data points are presented as Mean ± SEM.

Supplemental Figure 2.

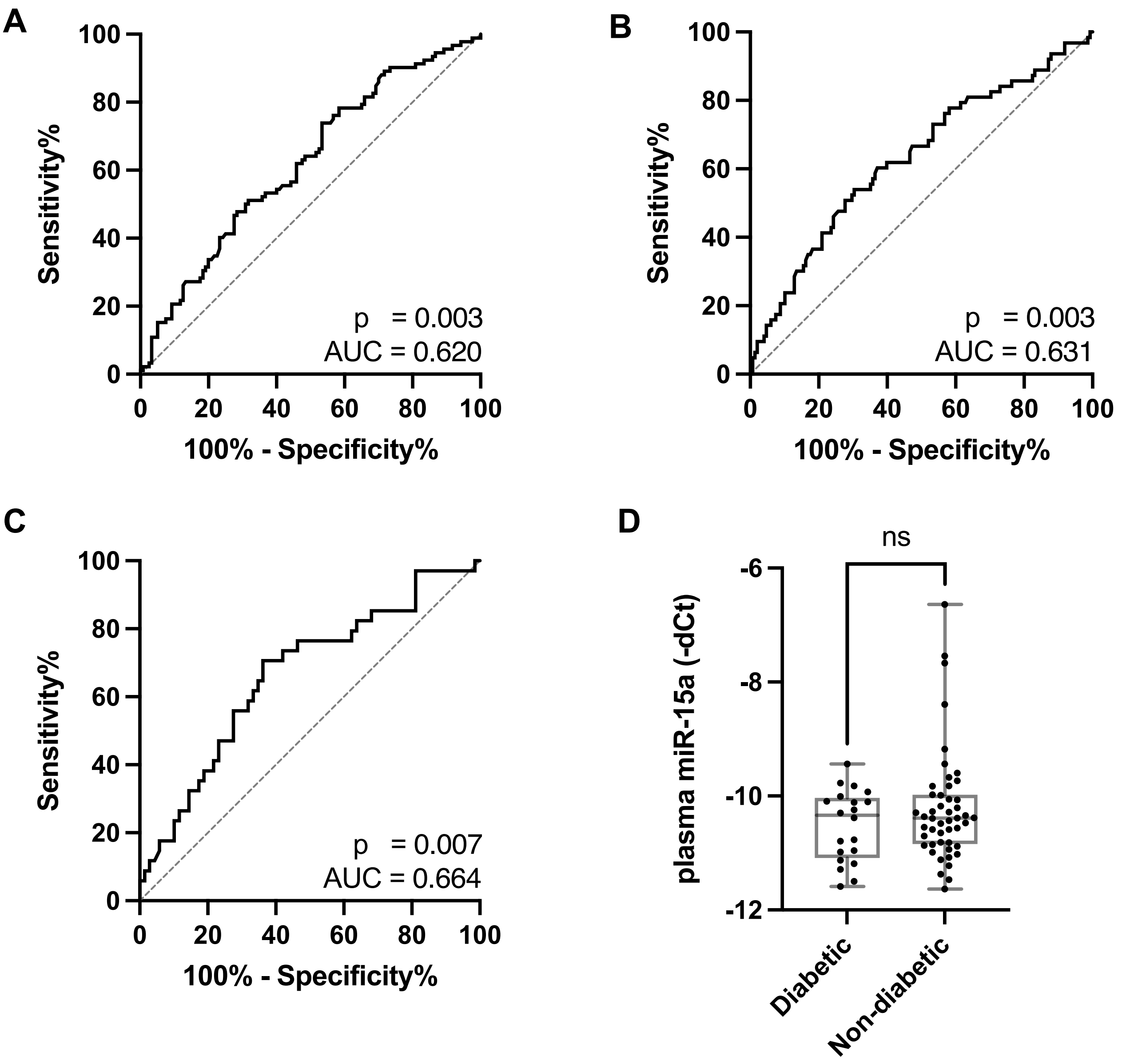

A & B. Receiver operating characteristic (ROC) curve for data in Figure 1C. AAA vs controls in **(A)**, AAA >50 mm vs controls and AAA ≤50mm in **(B)**. P-values for AUC > 0.5. **C.** ROC curve for data in Figure 2E, AAA ≥40mm vs controls and AAA ≤39mm. P-values for AUC > 0.5. **D.** Expression of miR-15a in plasma of AAA patients (Stockholm AAA screening cohort; n=68) with or without type 2 diabetes.

Supplemental Figure 3.

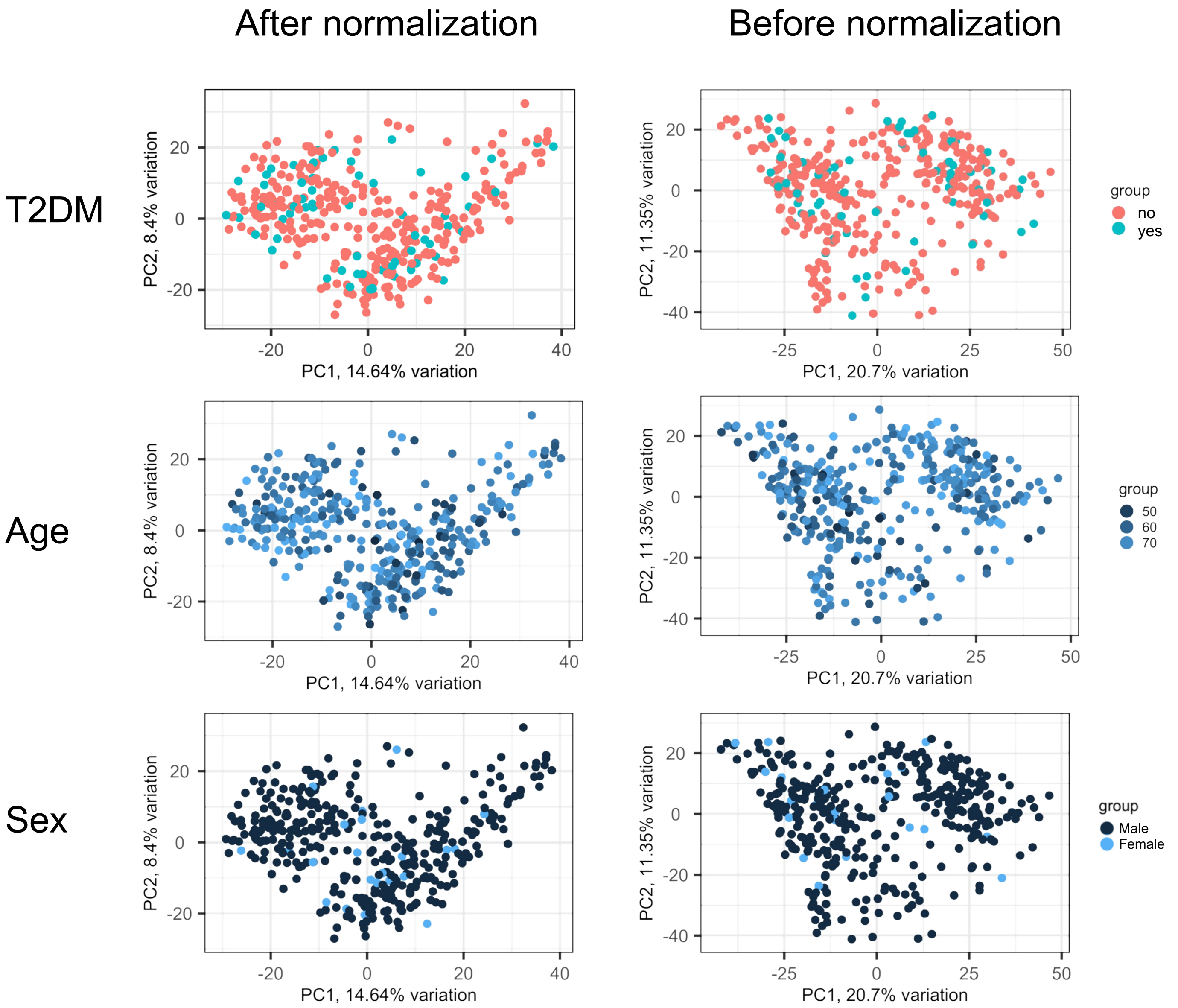

Principal component analysis of miRNA expression in 377 plasma samples analysed through OpenArray (SMART cohort).

Supplemental Figure 4.

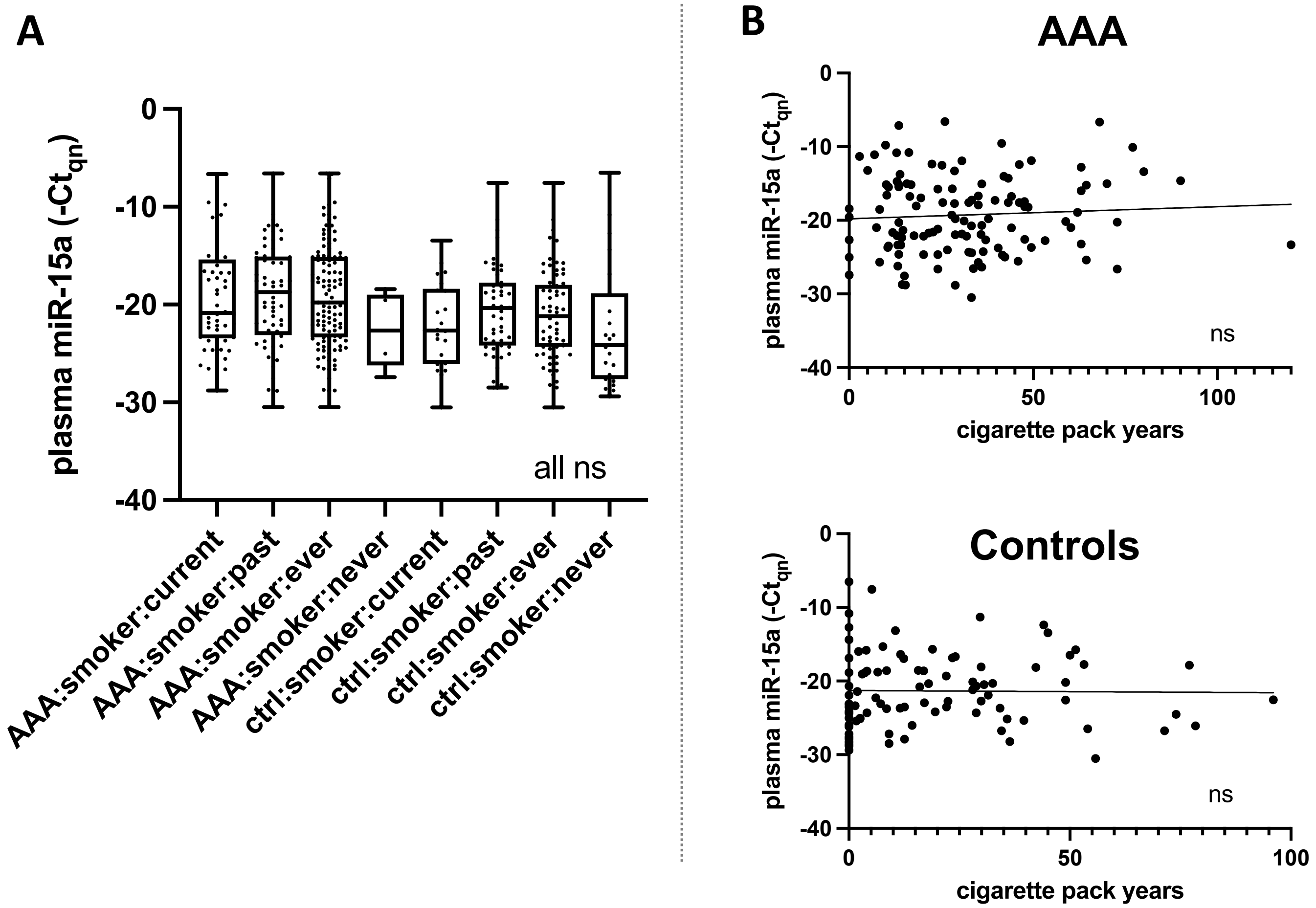

A. Quantile normalized Ct values (Ct_{qn}) of miR-15a in SMART AAA patients – current smokers (n=54), past smokers (n=60), ever smokers (n=114), never smokers (n=5) and control patients – current smokers (n=22), past smokers (n=47), ever smokers (n=69), never smokers (n=23). **B.** Correlation of plasma miR-15a expression with cigarette pack years in either AAA or control patients. Differences between means were analysed using unpaired Student's t-test. For analysis of correlation Pearson correlation coefficient (*r*) was calculated together with a p-value for linear regression being non-zero.

Supplemental Figure 5.

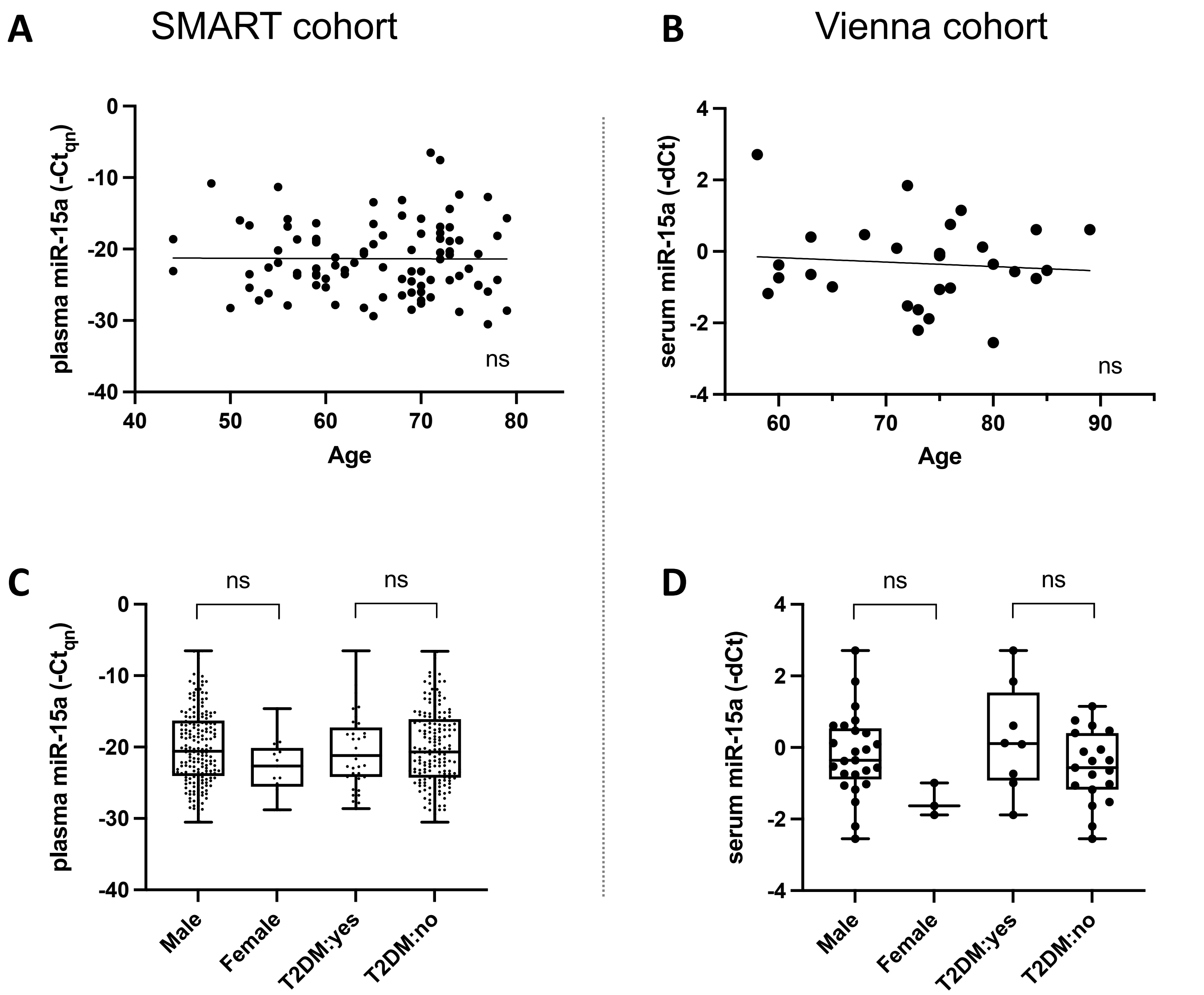

A & B. Correlation of plasma/serum miR-15a expression with patient age in SMART (A) and Vienna (B) cohorts. **C & D.** Quantile normalized Ct values (Ct_{qn}) of miR-15a in SMART (C) and Vienna (D) cohorts. Differences between means were analysed using unpaired Student's t-test. For analysis of correlation Pearson correlation coefficient (*r*) was calculated together with a p-value for linear regression being non-zero.

Supplemental Table 1. List of reagents

Antibodies	Manufacturer (cat. nr.)	Concentration
anti-a-SMA	Abcam (ab5694)	1:200
anti-rabbit Alexa 555	Thermofisher (A32732)	1:1000
anti-DIG-POD	Roche (11207733910)	1:400
**qPCR-primers**	**Manufacturer (cat. nr.)**	**Sequence**
hsa/mmu-miR-15a-5p	Thermofisher TaqMan (000389)	UAGCAGCACAUAAUGGUUUGUG
hsa/mmu-miR-192	Thermofisher TaqMan (000491)	CUGACCUAUGAAUUGACAGCC
hsa-miR-659	Thermofisher TaqMan (001514)	CUUGGUUCAGGGAGGGUCCCCA
hsa-miR-1183	Thermofisher TaqMan (002841)	CACUGUAGGUGAUGGUGAGAGUGGGCA
cel-miR-39	Thermofisher TaqMan (000200)	UCACCGGGUGUAAAUCAGCUUG
U6 snRNA	Thermofisher TaqMan (001973)	GTGCTCGCTTCGGCAGCACATATACTAAAATTGGAACGATA CAGAGAAGATTAGCATGGCCCCTGCGCAAGGATGACACGC AAATTCGTGAAGCGTTCCATATTTT
**miRNA-modulators (*in vitro*)**	**Manufacturer (cat. nr.)**	**Sequence**
hsa-miR-15a-5p mimic	Thermofisher mirVana (MC10235)	UAGCAGCACAUAAUGGUUUGUG
hsa-miR-15-5p inhibitor	Thermofisher mirVana (MH10235)	UAGCAGCACAUAAUGGUUUGUG
miRNA mimic neg ctrl	Thermofisher mirVana (4464058)	-
miRNA inhibitor neg ctrl	Thermofisher mirVana (4464076)	-
**miRNA-modulators (*in vivo*)**	**Manufacturer (cat. nr.)**	**Sequence**
mmu-miR-15a-5p inhibitor	Qiagen miRCURY (YCI0202576-FZA)	ACCATTATGTGCTGCT
miRNA inhibitor neg ctrl	Qiagen miRCURY (YCI0200994-FZA)	ACGTCTATACGCCCA
**ISH LNA probes**	**Manufacturer (cat. nr.)**	**Sequence**
hsa-miR-15a LNA Detection probe	Qiagen miRCURY Double-DIG labeled (YD00611553-BCG)	CACAAACCATTATGTGCTGCTA
Scrambled LNA Detection probe	Qiagen miRCURY Double-DIG labelled	GTGTAACACGTCTATACGCCCA
