# Supplementary material for: MicroRNA-15a-5p mediates abdominal aortic aneurysm progression and serves as a potential diagnostic and prognostic circulating biomarker
Source: Commun Med (Lond). 2025 Jun 6;5:218. doi: 10.1038/s43856-025-00892-w (PMC12144292; doi:10.1038/s43856-025-00892-w)
Supplement: Supplementary file 3 — Description of Additional Supplementary files [file 43856_2025_892_MOESM3_ESM.pdf]

Description of Supplementary Materials

File name: Supplementary Data 1

Supplementary Data 1 is the source data for Figures 1A-C.

Supplementary Data 2

Supplementary Data 2 are the source data for Figures 4-5.
