# Supplementary material for: MicroRNA-15a-5p mediates abdominal aortic aneurysm progression and serves as a potential diagnostic and prognostic circulating biomarker
Source: Commun Med (Lond). 2025 Jun 6;5:218. doi: 10.1038/s43856-025-00892-w (PMC12144292; doi:10.1038/s43856-025-00892-w)
Supplement: Supplementary file 6 — REPORTING SUMMARY [file 43856_2025_892_MOESM6_ESM.pdf]

nature portfolio

Corresponding author(s): Lars Maegdefessel

Last updated by author(s): Apr 11, 2024

Reporting Summary

Nature Portfolio wishes to improve the reproducibility of the work that we publish. This form provides structure for consistency and transparency in reporting. For further information on Nature Portfolio policies, see our Editorial Policies and the Editorial Policy Checklist.

Statistics

For all statistical analyses, confirm that the following items are present in the figure legend, table legend, main text, or Methods section.

n/a	Confirmed	
☐	☒	The exact sample size (*n*) for each experimental group/condition, given as a discrete number and unit of measurement
☐	☒	A statement on whether measurements were taken from distinct samples or whether the same sample was measured repeatedly
☐	☒	The statistical test(s) used AND whether they are one- or two-sided<br>*Only common tests should be described solely by name; describe more complex techniques in the Methods section.*
☒	☐	A description of all covariates tested
☐	☒	A description of any assumptions or corrections, such as tests of normality and adjustment for multiple comparisons
☐	☒	A full description of the statistical parameters including central tendency (e.g. means) or other basic estimates (e.g. regression coefficient) AND variation (e.g. standard deviation) or associated estimates of uncertainty (e.g. confidence intervals)
☐	☒	For null hypothesis testing, the test statistic (e.g. *F*, *t*, *r*) with confidence intervals, effect sizes, degrees of freedom and *P* value noted<br>*Give P values as exact values whenever suitable.*
☒	☐	For Bayesian analysis, information on the choice of priors and Markov chain Monte Carlo settings
☒	☐	For hierarchical and complex designs, identification of the appropriate level for tests and full reporting of outcomes
☐	☒	Estimates of effect sizes (e.g. Cohen's *d*, Pearson's *r*), indicating how they were calculated

Our web collection on statistics for biologists contains articles on many of the points above.

Software and code

Policy information about availability of computer code

Data collection	No software was used for data collection.
Data analysis	Hisat2 v2.0.5<br>FeatureCounts v1.5.0-p3<br>DESeq2 1.20.0<br>HTqPCR 1.44.0<br>limma 3.46<br>clusterProfiler 3.18.1

For manuscripts utilizing custom algorithms or software that are central to the research but not yet described in published literature, software must be made available to editors and reviewers. We strongly encourage code deposition in a community repository (e.g. GitHub). See the Nature Portfolio guidelines for submitting code & software for further information.

Data

Policy information about availability of data

All manuscripts must include a data availability statement. This statement should provide the following information, where applicable:

- Accession codes, unique identifiers, or web links for publicly available datasets
- A description of any restrictions on data availability
- For clinical datasets or third party data, please ensure that the statement adheres to our policy

Key data from the analyses in this article is available in the online supplementary material and all remaining data will be shared on reasonable request to the corresponding author. RNAseq data generated as part of this manuscript will be deposited in the NCBI Gene Expression Omnibus (GEO).

Research involving human participants, their data, or biological material

Policy information about studies with human participants or human data. See also policy information about sex, gender (identity/presentation), and sexual orientation and race, ethnicity and racism.

Reporting on sex and gender	Not applicable as no patient recruitment was performed for this study, only retrospective analysis of existing patient cohorts
Reporting on race, ethnicity, or other socially relevant groupings	Not applicable as no patient recruitment was performed for this study, only retrospective analysis of existing patient cohorts
Population characteristics	Not applicable as no patient recruitment was performed for this study, only retrospective analysis of existing patient cohorts
Recruitment	Not applicable as no patient recruitment was performed for this study, only retrospective analysis of existing patient cohorts
Ethics oversight	All collection of human materials was approved by the respective local ethic committees (Stockholm 00-337, 2005/83-31, 2009/9-31/4, 2011/1863-32, 2012/916-31/4, 2018/176-32, 2019-04561, 2019-05050, 2019-05051; Munich 2799/10; Utrecht 13-579; Vienna 1729/2014) and followed the guidelines of the Declaration of Helsinki. All human samples and data were collected after obtaining informed consent.

Note that full information on the approval of the study protocol must also be provided in the manuscript.

Field-specific reporting

Please select the one below that is the best fit for your research. If you are not sure, read the appropriate sections before making your selection.

☒ Life sciences ☐ Behavioural & social sciences ☐ Ecological, evolutionary & environmental sciences

For a reference copy of the document with all sections, see nature.com/documents/nr-reporting-summary-flat.pdf

Life sciences study design

All studies must disclose on these points even when the disclosure is negative.

Sample size	SMART cohort: Based on calculations by the PASS software package (Department of Biostatistics, College of Public Health, UNMC, Omaha, NE), we calculated that a sample size of 200 cases (patients with an abdominal aortic diameter = AAD at baseline of >3cm) and 150 controls (AAD <3cm) was needed to achieve 96% power for each miRNA, to detect a true difference in expression of at least 0.1 with estimated group standard deviations of 0.1 and 0.1 and with an experiment-wise error rate (EWER) of 0.0500 using a two-sided two-sample t test.<br><br>Vienna AAA cohort: no sample size was calculated, all material made available from collaborators was used.<br><br>Stockholm screening cohort: no sample size was calculated, all material made available from collaborators was used.
Data exclusions	OpenArray: Features that could not be successfully detected in more than 50% of the samples were excluded. From 203 AAA patient plasma samples and 203 matched controlled samples, 16 and 13 respectively were of insufficient quality or quantity to be analyzed. No supervised exclusions were made.
Replication	The hypotheses were tested across multiple patient cohorts and experiment types (experimental in vitro and in vivo) in order to verify validity and reproducibility.
Randomization	Not relevant as no prospective experimental design was present.
Blinding	This is only applicable to analysis of experimental murine AAA diameter measurements. Measurements were made by an independent researcher otherwise not involved in performing the animal interventions, without access to information on which treatment they had received.

Reporting for specific materials, systems and methods

We require information from authors about some types of materials, experimental systems and methods used in many studies. Here, indicate whether each material, system or method listed is relevant to your study. If you are not sure if a list item applies to your research, read the appropriate section before selecting a response.

Materials & experimental systems

n/a	Involved in the study
☐	☒ Antibodies
☒	☐ Eukaryotic cell lines
☒	☐ Palaeontology and archaeology
☐	☒ Animals and other organisms
☒	☐ Clinical data
☒	☐ Dual use research of concern
☒	☐ Plants

Methods

n/a	Involved in the study
☒	☐ ChIP-seq
☒	☐ Flow cytometry
☒	☐ MRI-based neuroimaging

Antibodies

Antibodies used	anti-a-SMA (Abcam ab5694)<br>anti-rabbit Alexa 555 (Thermofisher A32732)<br>anti-DIG-POD (Roche 11207733910)
Validation	All three antibodies have been widely validated for use in mouse (ab5694, validated by manufacturer and a vast number of publications). The remaining 2 are widely used as secondary antibodies.

Animals and other research organisms

Policy information about studies involving animals; ARRIVE guidelines recommended for reporting animal research, and Sex and Gender in Research

Laboratory animals	AngII infusion AAA model: 10-week-old male ApoE-/- mice (Taconic Biosciences, Hudson, NY, USA)<br>PPE model: 10-week-old male C57BL/6 mice (Charles River, Wilmington, MA, USA)
Wild animals	Not applicable
Reporting on sex	Only male mice were used in the experiments as the models have not been widely validated on female mice. This should be considered a potential limitation for application of results onto female AAA disease.
Field-collected samples	Not applicable
Ethics oversight	Animal procedures conformed with EU and Swedish legislation concerning the protection of animals used for scientific purposes, and were performed under ethical approval of the local ethics committee (Swedish Board for Agriculture; Ethical permit no. N48/16).

Note that full information on the approval of the study protocol must also be provided in the manuscript.

Plants

Seed stocks	Not applicable
Novel plant genotypes	Not applicable
Authentication	Not applicable
